# Supplementary material for: A Multifaceted Intervention and Its Effects on Antibiotic Usage in Norwegian Nursing Homes
Source: Antibiotics (Basel). 2023 Aug 27;12(9):1372. doi: 10.3390/antibiotics12091372 (PMC10526029; doi:10.3390/antibiotics12091372)
Supplement: Supplementary file 1 [file antibiotics-12-01372-s001.zip › antibiotics-2525490-supplementary.pdf]

Table S1. Total mean antibiotic use in DDD/100 BD, and the model-based * estimated changes identified ** pre- (Oct. 2015 – Oct. 2016) and post-intervention (Oct. 2016 – Oct. 2017) for the control counties.

Counties	DDD/100 BD Baseline	DDD/100 BD Intervention	β	95% CI	P
Buskerud (control)	7.48	7.28	-0.23	-1.09 – 0.63	0.604
Agder (control)	7.01	7.63	0.61	-0.11 – 1.34	0.098
Telemark (control)	11.68	10.24	-1.54	-3.12 – 0.05	0.057
Vestfold (control)	9.21	7.34	-1.84	-3.39 – -0.29	0.02

* Linear mixed model regression analysis.

** All models adjusted for NH size, NH category and doctor hours/bed/week.

Figure S1. Changes in antibiotic use in DDD/100 BD per nursing home category and per county, pre- and post-intervention periods, for the intervention county and control counties, Oct. 2015 – Oct. 2016 versus Oct. 2016 – Oct. 2017

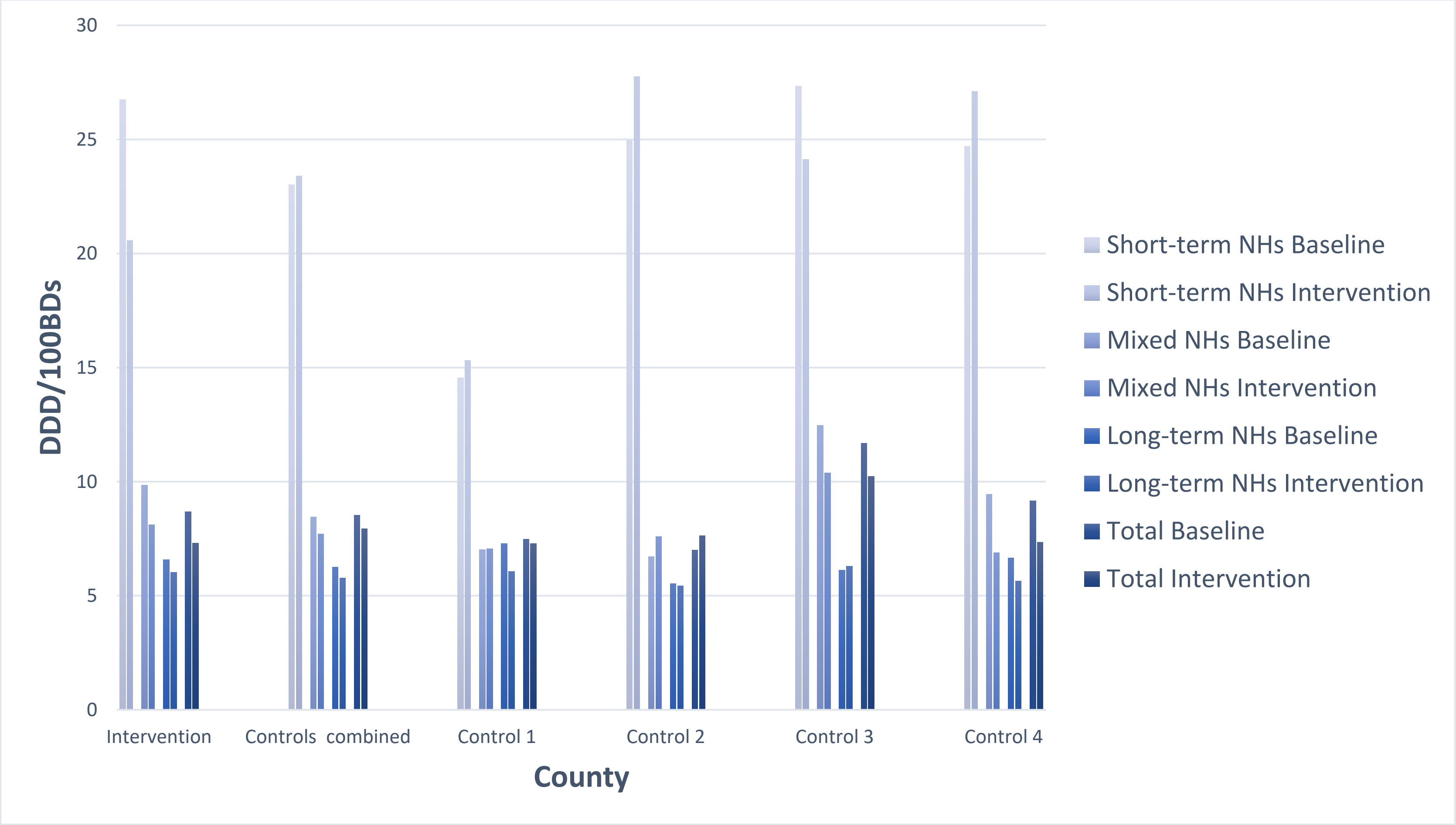

Table S2. Linear mixed model to identify the model-based comparison of changes in proportion of guideline-compliant antibiotics, pre- (Oct. 2015 – Oct. 2016) versus post-intervention (Oct. 2016 – Oct. 2017) between the intervention county and the control counties combined (number of observations: 336).

	Estimated Difference β	95% CI	P
Change in guideline-compliant antibiotic use in control counties combined	ref.		
Comparison of change in guideline-compliant antibiotic use between intervention and controls combined	-0.03	-0.09 – 0.02	0.23
Nursing home category:			
Mixed	ref.		
Long-term	-0.01	-0.06 – 0.04	0.71
Short-term	-0.06	-0.13 – 0.003	0.06
Size of nursing home:			
Small	ref.		
Medium	-0.001	-0.04 – 0.04	0.96
Large	-0.08	-0.15 – 0.004	0.04
Doctor hours/bed/week	< 0.001	< -0.001 – 0.001	0.2

Table S3. Linear mixed model to evaluate the model-based estimated change in total methenamine use, pre- (Oct. 2015 – Oct. 2016) versus post-intervention (Oct. 2016 – Oct. 2017) in the intervention county (number of observations: 321).

	Estimated Change		
	β	95% CI	P
Change in total methenamine use in the intervention county	-1.34	-2.24 – -0.44	0.003
Nursing home category:			
Mixed	ref.		
Long-term	1.47	-1.09 – 4.03	0.26
Short-term	-2.9	-6.09 – 0.3	0.08
Size of nursing home:			
Small	ref.		
Medium	-1.83	-4.18 – 0.51	0.13
Large	-0.83	-4.32 – 2.67	0.64
Doctor hours/bed/week	-0.02	-0.06 – 0.02	0.27

Table S4. Linear mixed model regression analysis to evaluate the model-based estimated comparison of change in methenamine use, pre- (Oct. 2015 – Oct. 2016) versus post-intervention (Oct. 2016 – Oct. 2017) between the intervention county and control counties (number of observations: 336).

	Estimated Difference β	95% CI	P
Change in total methenamine use in control counties combined	ref.		
Comparison of change in total methenamine use between intervention and combined controls	-1.34	-2.55 – -0.14	0.029
Nursing home category:			
Mixed	ref.		
Long-term	1.57	-1.08 – 4.21	0.245
Short-term	-2.01	4.71 – 0.69	0.145
Size of nursing home:			
Small	ref.		
Medium	-1.33	-3.81 – 1.14	0.292
Large	-0.01	-3.26 – 3.24	0.997
Doctor hours/bed/week	-0.03	-0.07 – 0.01	0.106
